# Occurrence and genotyping of *Enterocytozoon bieneusi* in flying squirrels (*Trogopterus xanthipes*) from China[Fn FN1]

**DOI:** 10.1051/parasite/2024037

**Published:** 2024-07-04

**Authors:** Xuehan Liu, Chi Zhang, Tiantian Li, Xiaojing Xia, Yanzhao Xu, Jianhe Hu, Longxian Zhang, Lei Wang, Meng Qi

**Affiliations:** 1 College of Animal Science and Veterinary Medicine, Henan Institute of Science and Technology Xinxiang 453003 Henan PR China; 2 Postdoctoral Research Base, College of Veterinary Medicine, Henan Agricultural University Zhengzhou 450046 PR China; 3 College of Animal Science and Technology, Tarim University Alar 843300 Xinjiang PR China

**Keywords:** *Enterocytozoon bieneusi*, Genotype, Zoonotic, ITS gene, Flying squirrel, Faeces trogopterori

## Abstract

*Enterocytozoon bieneusi* is an obligate intracellular microsporidian parasite with a worldwide distribution. As a zoonotic pathogen, *E. bieneusi* can infect a wide range of wildlife hosts through the fecal-oral route. Although the feces of flying squirrels (*Trogopterus xanthipes*) are considered a traditional Chinese medicine (as “faeces trogopterori”), no literature is available on *E. bieneusi* infection in flying squirrels to date. In this study, a total of 340 fresh flying squirrel fecal specimens from two captive populations were collected in Pingdingshan city, China, to detect the prevalence of *E. bieneusi* and assess their zoonotic potential. By nested PCR amplification of the ITS gene, six specimens tested positive, with positive samples from each farm, with an overall low infection rate of 1.8%. The ITS sequences revealed three genotypes, including known genotype D and two novel genotypes, HNFS01 and HNFS02. Genotype HNFS01 was the most prevalent (4/6, 66.7%). Phylogenetic analysis showed that all genotypes clustered into zoonotic Group 1, with the novel genotypes clustering into different subgroups. To our knowledge, this is the first report of *E. bieneusi* infection in flying squirrels, suggesting that flying squirrels could act as a potential reservoir and zoonotic threat for *E. bieneusi* transmission to humans in China.

## Abstract (fr)

*Enterocytozoon bieneusi* est un parasite microsporidien intracellulaire obligatoire présent dans le monde entier. En tant qu’agent pathogène zoonotique, *E. bieneusi* peut infecter un large éventail d’hôtes sauvages par la voie fécale-orale. Bien que les excréments d’écureuils volants (*Trogopterus xanthipes*) soient considérés comme un ingrédient de médecine traditionnelle chinoise (comme « faeces trogopterori »), aucune littérature n’est disponible à ce jour sur l’infection par *E. bieneusi* chez les écureuils volants. Dans cette étude, un total de 340 spécimens fécaux frais d’écureuils volants provenant de deux populations captives ont été collectés dans la ville de Pingdingshan, en Chine, pour détecter la prévalence d’*E. bieneusi* et évaluer leur potentiel zoonotique. Par amplification PCR nichée du gène ITS, six échantillons se sont révélés positifs, avec des échantillons positifs dans chaque ferme, et un taux d’infection global faible, à 1,8 %. Les séquences ITS ont révélé trois génotypes, dont le génotype D connu et deux nouveaux génotypes, HNFS01 et HNFS02. Le génotype HNFS01 était le plus répandu (4/6, 66,7 %). L’analyse phylogénétique a montré que tous les génotypes se regroupaient dans le groupe zoonotique 1, les nouveaux génotypes se regroupant en différents sous-groupes. À notre connaissance, il s’agit du premier rapport d’infection par *E. bieneusi* chez des écureuils volants, ce qui suggère que les écureuils volants pourraient agir comme un réservoir potentiel et une menace zoonotique pour la transmission d’*E. bieneusi* aux humains en Chine.

## Introduction

Microsporidia are opportunistic zoonotic parasites that comprise a highly diverse group of obligate intracellular fungi [15]. Currently, over 1500 microsporidian species from more than 200 genera have been described, of which 17 species infect humans [14]. *Enterocytozoon bieneusi*, as an important emerging pathogen, is the most frequently detected species in human microsporidiosis worldwide (responsible for >90% of cases) and is involved in food, waterborne, and hospital-related disease outbreaks [7, 16]. In addition, more than 240 animal species, including vertebrates and invertebrates, can be infected with *E. bieneusi* [10, 29]. Recently, vegetables and fruits were verified as carriers of *E. bieneusi*, posing a public health concern [11]. In general, the pathogen is primarily transmitted through direct oral-fecal contact, whereas ingestion of spore-contaminated water or food also facilitates infection with *E. bieneusi* [15]. The clinical symptoms of *E. bieneusi* infection in immune-deficient persons include chronic or fatal diarrhea, malnutrition, weight loss, vomiting, dysentery, fever, cholangitis, rhinitis, pulmonary involvement, and other diseases, but in immunocompetent individuals, asymptomatic and self-limiting diarrhea are common [7, 12, 14, 29]. Similarly, infections commonly appear to be asymptomatic in animal hosts [15].

At present, PCR-based detection is universally acknowledged as the standard diagnostic method for *E. bieneusi* infection. The identification of this parasite is accomplished by sequence analysis of the internal transcribed spacer (ITS) region of the nuclear ribosomal DNA gene, and sequences have revealed extensive genetic diversity [14]. Globally, at least 920 genotypes have been recorded to date from various sources, including human, animal, food, water, and environment samples, from more than 50 countries. Of these, nearly 700 valid genotypes were identified with the accurate typing definition of *E. bieneusi* [1, 4, 5, 9, 10, 12, 14, 18, 22, 24, 25, 33, 34, 36, 38, 40]. Additionally, comprehensive phylogenetic analysis has classified these ITS genotypes into 14 distinct genetic clusters, named Group 1–14 [25, 39]. The genotypes in Group 1 are further divided into nine subgroups (1a–1i) and infect both humans and animals, posing a zoonotic threat [19]. The genotypes in the remaining genetic groups (2–14) were primarily isolated from specific hosts, suggesting strong host specificity and a limited public health threat [15, 25, 39]. There are exceptions, however, with a few genotypes from Group 2 (such as BEB4, BEB6, I, and J), isolated from ruminants, being zoonotic [14]. Excitingly, in the last few years, China has excelled in reporting novel *E. bieneusi* genotypes, with over 500 recorded by the end of December 2023 [4, 9, 10, 12, 18, 25, 30, 33, 34, 36, 38, 40]. Studies detecting zoonotic genotypes in animal fecal samples have also elucidated the transmission mechanism of *E. bieneusi* between animals and humans [21]. However, the transmission mechanism of *E. bieneusi* from specific animals to humans remains unclear.

Currently, wild animals including rodents, are considered the largest reservoirs of *E. bieneusi* (>640 genotypes and >75 zoonotic) and act as vehicles for the initiation of human microsporidiosis [1, 9, 10, 25, 26, 32, 34, 36, 38]. Thus, understanding the source and transmission mechanism of *E. bieneusi* in wildlife will help prevent its spread [16, 29]. In China, the feces of flying squirrels are considered a kind of traditional Chinese medicine named “faeces trogopterori” (Wulingzhi). The feces are collected manually by culturists and drunk by patients, which could facilitate zoonotic infection. Thus, the objective of this study was to determine the prevalence of *E. bieneusi* in the feces of medicinal flying squirrels from China, and to explore the diversity of genotypes as well as evaluate the zoonotic risk of the isolates.

## Material and methods

### Ethics statement

This study was reviewed and approved by the Ethics Committee of Henan Institute of Science and Technology (No. LLSC2022045). Permissions were obtained from farm owners before sampling, and no animals were harmed during the collection of fecal samples.

### Specimen collection

A total of 340 fresh flying squirrel fecal specimens were collected from two farms in Pingdingshan city, central China, from 2019 to 2021 (Table 1). Each fresh fecal sample of ~20 g each was collected from the bottom of the cage with a new plastic bag to avoid contamination and cross-contamination. The available farm information was recorded on the bag at the time of sampling. All fecal specimens collected were put into a cryopreservation box with enough ice bags and transported to the laboratory for storage at 4 °C within 24 h. Sample DNA was extracted immediately upon arrival at the laboratory.

**Table 1:** Prevalence and genotype of *Enterocytozoon bieneusi* infection in flying squirrels in China.

Farm	No. specimens	No. positive	Prevalence	*p*	Genotype
1	148	3	2%	0.747	D(1), HNFS01(1), HNFS02(1)
2	192	3	1.6%	Reference	HNFS01(3)
Total	340	6	1.8%		D(1), HNFS01(4), HNFS02(1)

### DNA extraction and PCR amplification

Approximately 200 mg of each specimen was used for genomic DNA extraction using commercial E.Z.N.A^®^ Stool DNA kits (Omega Bio-tek Inc., Norcross, GA, USA), following the manufacturer’s instruction. All DNA specimens were stored at −20 °C prior to molecular assays.

*Enterocytozoon bieneusi* infection was diagnosed using nested PCR amplification of a 390 bp fragment of the ITS region of the rDNA gene. The primers and PCR conditions used here were described previously [13]. 2×EasyTaq PCR SuperMix (TransGene Biotech Co., Beijing, China) was used for PCR reactions, and 2 μL of extracted DNA or primary PCR products were used as templates. In addition, 2 μL ddH_2_O and DNA of the *E. bieneusi* genotype D isolated from a bamboo rat were included in each PCR analysis as a negative and positive control, respectively. All final PCR amplicons were visualized with electrophoresis on a 1% agarose gel with Golden View (Sangon, Shanghai, China) staining.

### Genotyping of *E. bieneusi*

All secondary positive PCR products were sent for Sanger Sequencing using an ABI730 Autosequencer (GENEWIZ, Suzhou, China). The accuracy of sequences was confirmed by performing bidirectional sequencing and replenishing PCR products. To identify the genotypes of the *E. bieneusi* isolates, the nucleotide sequences obtained from each specimen were compared with published genotype sequences using a BLAST search (https://blast.ncbi.nlm.nih.gov/Blast.cgi). Sequences were then aligned with each other and corresponding reference sequences downloaded from the GenBank database using ClustalX 2.1 (http://www.clustal.org/). Novel genotypes were verified by sequencing independent PCR products twice and by calculating the genetic difference of the 243 bp ITS region. The naming of novel genotypes followed the established nomenclature regulations [23]. Sequences identical to submitted sequences in the NCBI database were identified as known genotypes.

### Phylogenetic analysis

A neighbor-joining tree was constructed to assess the phylogenetic relationships of the novel and known genotypes of *E. bieneusi* using Mega X (http://www.megasoftware.net/) based on genetic distances calculated using the Kimura 2-parameter model. Then, 1000 bootstrap replicates were used to evaluate the robustness of the tree. Bootstrap values >50% are shown on the branches of the tree.

### Statistical analysis

Differences in the infection rate of *E. bieneusi* between farms were analyzed with a *χ*^2^ test in SPSS version 25.0 (IBM SPSS Inc., Chicago, IL, USA), and differences with *p* < 0.05 were considered significant.

### Nucleotide sequence accession numbers

The representative nucleotide sequences of the *E. bieneusi* ITS gene obtained in this study were submitted to GenBank under the following accession numbers: MK947105, PP309817, and PP309818.

## Results and discussion

To our knowledge, this study is the first to explore the infection status of *E. bieneusi* in flying squirrels. Of the 340 collected fecal specimens in the present study, six were positive for *E. bieneusi*, equaling a prevalence of 1.8% (Table 1), which is comparable with the infection rates in chipmunks (3.6%) and mice (1.1%), but lower than those of the majority of rodents surveyed [38]. Rodents are commonly infected with *E. bieneusi* and are considered important zoonotic hosts, with prevalences ranging from 1.1% to 87.5% [14, 38, 39]. More specifically, in this study, positive specimens were found on each farm, where the farm-specific infection rates for Farms 1 and 2 were 2% and 1.6%, respectively (Table 1).

The reason for the discrepant prevalences seen in studies is not known, but it could be because of management, farming style, living conditions, and food sources (Table 2) [16]. For the Sciuridae specifically, the present study revealed infection rates similar to the Alashan ground squirrel (3%) and Pallas’s squirrel (4.2%) from Gansu and Hainan in China, respectively [31, 39], but significantly lower than that of the Red squirrel from Sichuan province and the Eastern gray squirrel from the USA (Table 2) [2, 6]. Therefore, the differences in infection rates within the Sciuridae hosts had no relation to species. Furthermore, because cypress leaves and seeds are the dominant food sources of flying squirrels, faeces trogopterori has been verified to contain insecticidal and antibacterial ingredients, possibly leading to the low infection rate of *E. bieneusi* in these flying squirrels (http://www.360doc.com/content/13/0130/13/18452_263226787.shtml).

**Table 2:** Prevalence and distribution of *Enterocytozoon bieneusi* genotypes in Sciuridae in the different countries.

Hosts	Prevalence (No. positive/no. samples)	*p*	Genotypes	Group (Clustered)	Country	References
Eastern grey squirrel (*Sciurus carolinensis*)	32.4% (11/34)	0	**Type IV**, **WL21**, WL4, WW6, PtEBV	1, 3, 4	USA	[6]
Pallas’s squirrel (*Callosciurus erythraeus*)	4.2% (1/24)	0.408	**D**	1	Hainan, China	[39]
Pallas’s squirrel (*Callosciurus erythraeus*)	13% (55/423)	0	SCC-2	12	Japan	[20]
Pallas’s squirrel (*Callosciurus erythraeus*)	16.7% (24/144)	0	**D**, **EbpC**, SC02, CE01, CE02	1, 6	Sichuan, China	[3]
Red squirrel *(Sciurus vulgaris*)	19.4% (61/314)	0	**D**, RS01, RS02, SCC-2, SCC-3	1, 12	Sichuan, China	[2]
Alashan ground squirrel (*Spermophilus alashanicus*)	3.0% (3/99)	0.434	HN39, HN96, YAK1	1	Gansu, China	[31]
Flying squirrel (*Trogopterus xanthipes*)	1.8% (6/340)	Reference	**D**, HNFS01, HNFS02	1	Henan, China	**This study**

Bold: known genotypes in previous studies.

**Figure 1:**
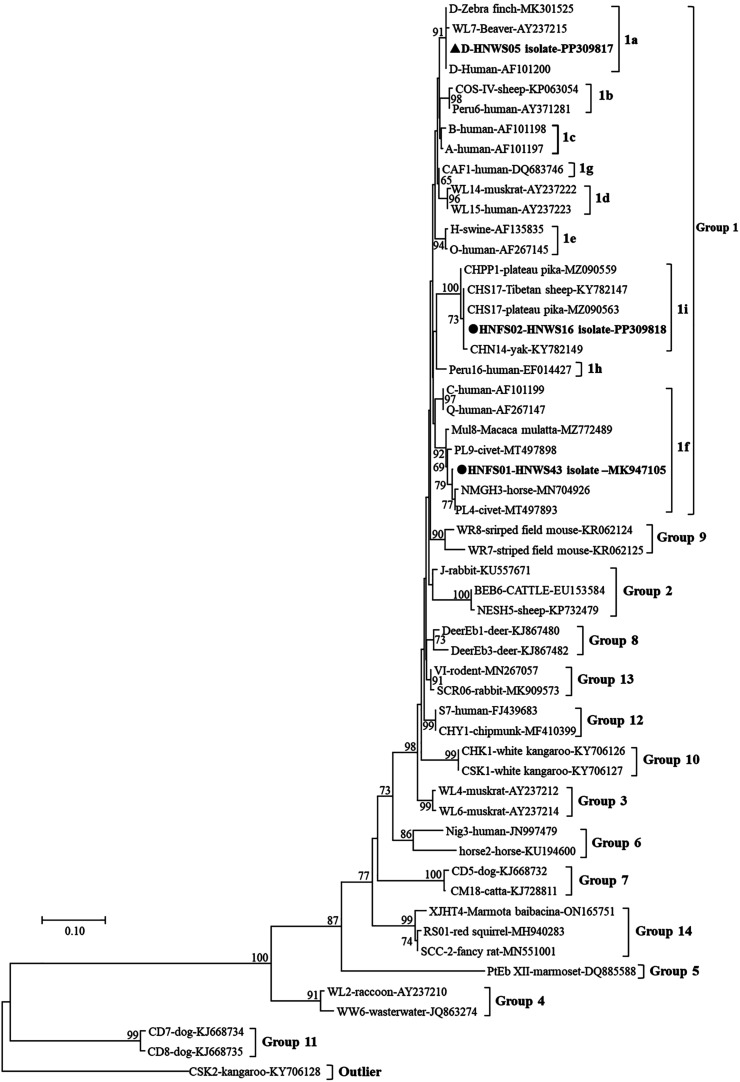
Phylogenetic relationships of *Enterocytozoon bieneusi* ITS gene isolates from flying squirrel samples in China, as inferred by the neighbor-joining (NJ) method based on evolutionary distances calculated using the Kimura two-parameter model. Bootstrap values were obtained using 1000 pseudoreplicates. Bar = substitutions/site; Triangle = known genotype; Circle = novel genotype.

Based on sequence analysis of the PCR products, three *E. bieneusi* genotypes were identified, including two novel genotypes, HNFS01 and HNFS02, and one known genotype D. All three genotypes were determined in samples from Farm 1 (Table 1), but only the HNFS01 genotype was found on Farm 2. The novel genotypes were isolated from five specimens (5/6, 83.3%), where the HNFS01 genotype had a 99.7% similarity to genotype PL4 (MT497893) with one nucleotide variation at position 69 (T-C) within the 243 bp section of the ITS sequence. Similarly, the HNFS02 genotype also revealed a single base difference at position 17 (T-C) compared with the CHS17 genotype (MZ090563). In the phylogenetic tree, all the genotypes clustered into Group 1, grouped into three different subgroups: 1a, 1f, and 1i (Fig. 1). As expected, genotype D from this study clustered with other known D genotype strains from various hosts (such as human, beaver, and zebra finch). In recent years, there has been a rapid increase in the number of *E. bieneusi* genotypes detected from wildlife in China, and hitherto, at least 130 genotypes have been identified from a variety of wild hosts, most of which are emerging genotypes [8, 9, 17, 25, 27–29, 32, 34, 35, 38, 39]. These records play an important role in exploring wild hosts of *E. bieneusi* worldwide, and the results of this study add to the growing genetic diversity and host range of *E. bieneusi* in wildlife.

Globally, many studies have shown that genotype D is the most prevalent zoonotic *E. bieneusi* in human, animal, and environmental specimens, having high public health importance and great cross-species potential [14, 16]. Although the zoonotic risk of genotype D in the present study is small, it should not be ignored. As the dominant strain of *E. bieneusi* in flying squirrels, the HNFS01 genotype assigned to zoonotic Group 1, also elicits a public health concern. Previous data showed that some genotypes in Group 1 (such as B, EbpB, and Peru10) display strong host-specificity and had small cross-species potential as inferred by their extremely restricted host range and population genetic analysis [14, 16, 37]. Since no surveys were conducted to determine the *E. bieneusi* infection in domestic animals and humans around these flying squirrel farms, the available data suggest that genotype HNFS01 is probably flying squirrel specific. As an added note, the emergence of genotype HNFS02 implies larger genetic diversity of *E. bieneusi* in the flying squirrel population.

**Figure 2:**
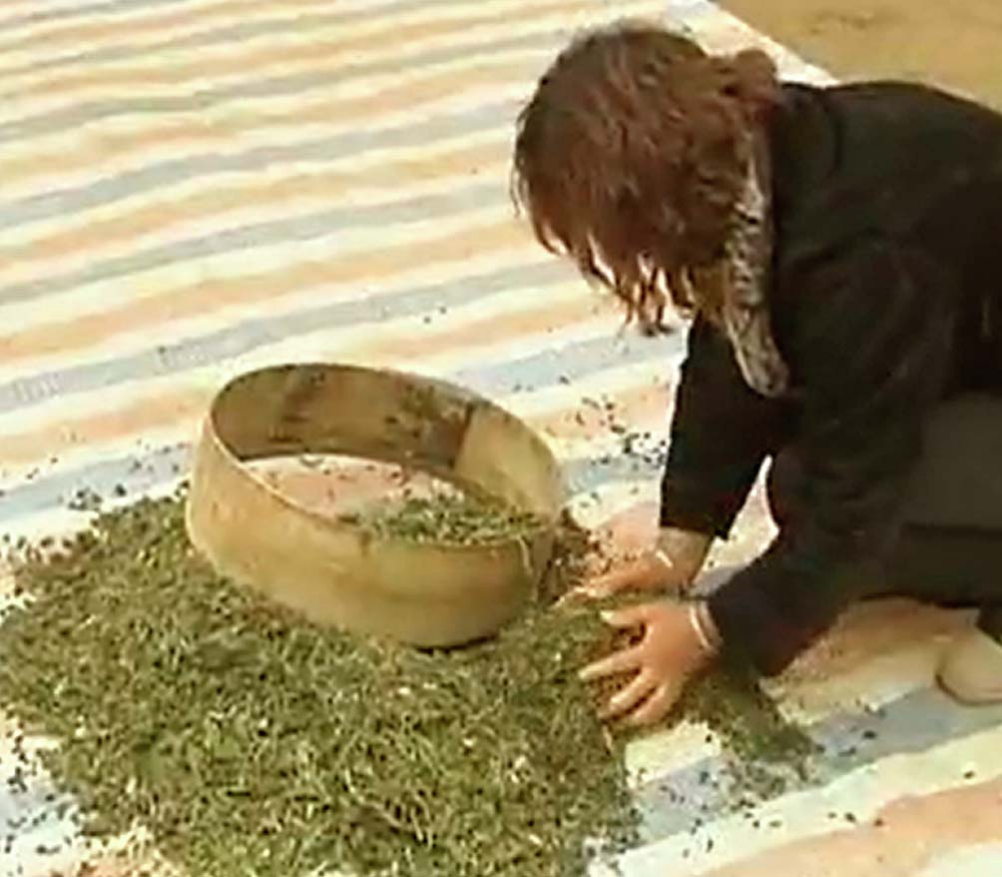
Manual screening of faeces trogopterori by a farmer.

**Figure 3:**
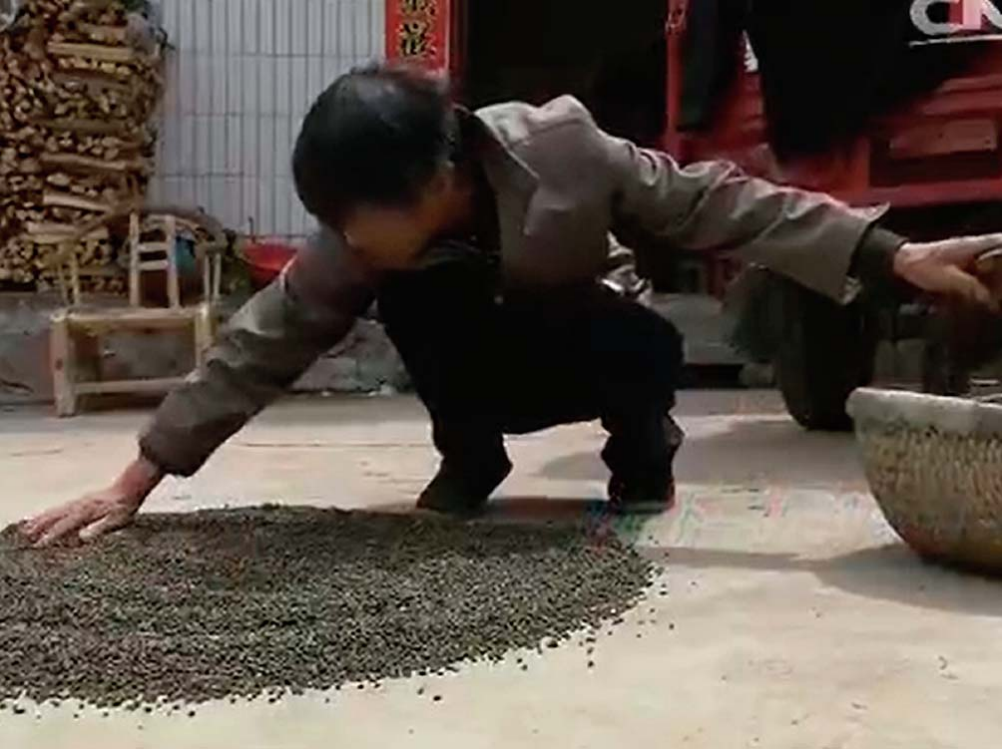
Manual drying of faeces trogopterori by a farmer.

To date, four squirrel species have been reported positive for *E. bieneusi* infection in five regions, with a total of 17 genotypes classified into five groups, including four known and 13 new genotypes (Table 2). Most of these belong to the zoonotic genotypes, suggesting that Sciuridae hosts can be important reservoirs of zoonotic *E. bieneusi* [2, 3, 6, 20, 31]. Faeces trogopterori is used to treat multiple human diseases via drinking of its decoction. The feces are collected manually, screened, dried and packaged by farmers, through corresponding procedures and storage of feces (https://baike.so.com/doc/5401113; Figs. 2 and 3). Thus, compared to studies of *E. bieneusi* in other wild animals, the repeated direct contact with flying squirrel feces on these farms poses an increased the risk of ‘fecal-oral’ transmission to humans, indicating a higher public health concern.

In conclusion, this study was the first to detect *E. bieneusi* infection in flying squirrels in China. A low prevalence of 1.8% was found, and three zoonotic genotypes were identified. These findings extend the host range, genetic diversity, and transmission potential of *E. bieneusi* in farmed medicinal wildlife. Even though novel genotypes were dominant, the genetic data, fecal characteristics (traditional Chinese medicine), and handling procedures revealed a clear zoonotic risk. Thus, additional studies in flying squirrels involving more specimens, regions, livestock, and communities near these farms and farmers are needed to further elucidate cross-species transmission and zoonotic threat.

## Data Availability

Data supporting the conclusions of this article are included in the article. Representative nucleotide sequences generated in this study were deposited in the GenBank database under accession numbers MK947105, PP309817, and PP309818.
